# Mixed methods to explore factors associated with the decline of patients in the methadone maintenance treatment program in Shanghai, China

**DOI:** 10.1186/s12954-019-0304-8

**Published:** 2019-05-27

**Authors:** Lei Zhang, Jiayi Bao, Amy Harrington, Xiaoduo Fan, Zhen Ning, Jingying Zhang, Daqing Shi, Manji Hu, Zhirong Zhou, Zhengyi Cai, Min Zhao, Jiang Du

**Affiliations:** 1 Department of Substance Abuse and Addiction, Shanghai Mental Health Center, Shanghai Jiao Tong University School of Medicine, Shanghai, China; 2 Psychotic Disorders Program, UMass Memorial Medical Center/University of Massachusetts Medical School, Worcester, MA USA; 3 Department of Public Health, Shanghai Municipal Center for Disease Control Prevention, Shanghai, China; 4 Shanghai Compulsory Drug Rehabilitation Center, Shanghai, China; 5 Department of Methadone Maintenance Treatment Clinics, Shanghai Yangpu Mental Health Center, Shanghai, China; 6 Department of Methadone Maintenance Treatment Clinics, Shanghai Xuhui Mental Health Center, Shanghai, China; 7 Department of Methadone Maintenance Treatment Clinics, Shanghai Pudong Mental Health Center, Shanghai, China; 8 Shanghai Key Laboratory of Psychotic Disorders, Shanghai, China; 9 Brain Science and Technology Research Center, Shanghai Jiao Tong University, Shanghai, China

**Keywords:** Heroin dependence, Methadone Maintenance Treatment, Dropout, Quantitative assessment, Qualitative assessment

## Abstract

**Background:**

This study was to characterize the Methadone Maintenance Treatment (MMT) in Shanghai, China, and to explore factors associated with the decline of patients in MMT during 2005–2016.

**Methods:**

Both qualitative and quantitative methods were used in this study. Based on the data from Shanghai Centers for Disease Control (CDC), we described the changes in the number of patients who received MMT, and new enrollment each year from 2005 to 2016. Focus groups were conducted with 22 patients, and in-depth interviews were conducted with 9 service providers.

**Results:**

Quantitative data demonstrate that the number of new enrollment began to decline in 2009, and the number of patients receiving MMT began to decline in 2012. The main reasons for dropout include (1) discontinuing medication due to unknown reasons (25%), (2) criminal activities other than drug-related crimes (20%), (3) relapse to heroin use (16%), and (4) physical disease (10%). Qualitative assessment results indicate that the major reasons for the decline of patients in MMT are as follows: (1) the increase of Amphetamine-type stimulants (ATS) use in recent years, (2) limited knowledge about MMT in both patients and MMT staff, (3) complicated enrollment criteria, and (4) discrimination against drug use.

**Conclusion:**

Various reasons to explain the decline of patients in MMT in Shanghai, China, were identified. Government agencies, service providers, and other stakeholders need to work together and overcome identified barriers to support MMT programs in China.

## Introduction

Opioid use disorder and other substance use disorders are major global health problems that severely impact communities and adversely affect both physical and mental health. China, given its large population, has been particularly vulnerable to negative consequences of the global opioid epidemic. The number of people with substance use disorders registered with the Chinese Public Security Bureau was 2.51 million in 2016, including 0.95 million heroin users; the actual number is estimated to be approximately 14 million [1]. In contrast, the estimated number of people with substance use disorders in 2002 was approximately 1 million [2]. In 2005, intravenous drug use contributed to 44.3% of new Human Immunodeficiency Virus (HIV) infections in China, making it the single largest cause of HIV transmission that year [3]. As a strategy for reducing high-risk behaviors associated with opioid use, the Chinese government began to implement the Methadone Maintenance Treatment (MMT) program which started in 2004, and rapidly scaled it up since 2007 when Anti-Drug Law of the People’s Republic of China was issued. By the end of 2016, there were 789 MMT clinics in the country serving 344,254 active patients [4]. Despite studies demonstrating the benefits of MMT in reducing the crime rate and preventing the transmission of HIV [5, 6], implementation of MMT in China continues to face numerous challenges.

The discrepancy between the number of registered substance users and the number of patients enrolled in MMT clinics indicates low utilization of this service, the only medication-assisted treatment currently available in China. In addition, the retention of individuals in MMT programs in China is low, ranging from 30.0 to 70.3% [7]. Studies in other countries showing similarly low retention rates of 20–85% have suggested that factors contributing to low retention include personality characteristics, gender, drug use history, social factors, and methadone dose [8]. Determining factors specific to low retention in Chinese programs could help facilitate the development and expansion of MMT services in China.

Shanghai, with a population of over 24 million, is considered the economic center of China. Fourteen MMT clinics have been established since 2005, serving approximately 7000 patients. The rate of people with substance use disorders registered with the Shanghai Municipal Security Bureau increases by approximately 10% each year. However, longitudinal data has not been maintained about the retention rate in MMT clinics, and there is currently no data on the trend of enrollment and retention in MMT clinics in China as few studies have sought to evaluate MMT programs in China. To address this knowledge gap, we used quantitative techniques to describe the variation in new enrollment in MMT programs from 2005 to 2016, and we used qualitative techniques to explore barriers to MMT retention in Shanghai. We hypothesized that there would be a decline in the number of MMT patients due to factors similar to those seen in other countries, including social factors and co-morbid drug use.

## Materials and methods

### Participants

#### Quantitative study

Data were obtained from the Shanghai Municipal Center for Disease Control (CDC) registry of patients receiving MMT. Data gathered include demographic information, methadone dose, and reason for termination of treatment.

#### Qualitative study

Participants in the qualitative study were recruited in July 2016 through advertising in Shanghai MMT clinics. All patients meet the requirements of National guidelines for MMT of drug dependence: (1) opioid users with more than one failed attempt at drug treatment, (2) at least 20 years of age, (3) a permanent local resident or a temporary resident lived in the area where the applicable MMT clinic is located for at least 6 months with a temporary resident permit, and (4) with legal capacity of decision making. Three groups of participants were interviewed. Two focus group discussions for current MMT patients were conducted, one for men and one for women. The third group was for participants who had dropped out of treatment. In-depth interviews were conducted with service providers (MMT staff, social workers, or administrators in MMT clinics or MMT related institutes) All of the prospective participants were informed that participation was voluntary, and for patients, refusal to participate would not affect their treatment.

The groups included patient participants, one facilitator, and two observers. Each focus group lasted approximately 2 h and addressed several topics: (1) knowledge and attitude about MMT, (2) treatment experience and demand of the patients with MMT, (3) reasons for termination of treatment, and (4) potential causes for the decline in MMT enrollment.

Informed consent for all participants occurred prior to each focus group or interview.

### Measures

#### Demographic characteristics

Demographics data were provided by the Shanghai Municipal Center for Disease Control registry and included the total number of patients receiving MMT and new enrollment from 2005 to 2016, the retention rate, the reasons for dropout, and the daily dosage of methadone.

Demographic information for participants in the qualitative study was obtained through a self-developed questionnaire and included age, gender, marital status, employment, and level of education.

#### Methadone Knowledge Scale (MKS)

The Methadone Knowledge Scale was developed by Caplehorn to test knowledge and cognition of risks and benefits of using methadone [9]. The MKS is a self-report scale, consisting of 35 items, each of which is answered “true,” “false,” or “I don’t know”. Respondents’ scores are calculated by adding “1” for a correct answer, “0” for “I don’t know” or incorrect answer. Thus, higher scores indicate greater knowledge and better cognition about MMT. This scale was shown to have acceptable test-retest reliability with staff working in methadone maintenance clinics [10]. All participants who participated in focus groups were required to complete this questionnaire.

#### Interview approach

Interviews with service providers and focus groups with MMT patients were conducted by experienced researchers in private rooms at MMT clinics. The coordinator explained the informed consent process and made sure each person understood the aim of the focus group discussions. Interviews and focus groups were both audio-recorded and also documented by hand-written notes by research assistants observing the discussions.

### Data analysis

Descriptive statistics were used to describe quantitative data including demographics, drug use history, MMT knowledge, and HIV knowledge. All statistical analyses were conducted with SPSS version 20.0.

Focus group notes were taken by two investigators and then entered into Microsoft Word. Qualitative data analysis was conducted by two researchers who read the transcribed group discussions separately to develop themes and then created a codebook that captured the textual themes using the interview guide questions as a framework. Interviews were then coded by two independent researchers to achieve an interpreter reliability of 80%. All coding disagreements were reconciled successfully.

The research protocol was approved by the Ethics Committee of the Shanghai Mental Health Center, and each subject signed the informed consent form approved by IRB at the Shanghai Mental Health Center.

## Result

### Quantitative study

Totally, 7181 clients received MMT between May 2005 and June 2016, and 77.9% of them were male. More than 50% of them were aged from 25 to 44-years old. Regarding the highest level of education, 65.4% of them had a middle school education or below. (Table 1)

**Table 1:** Demographic characteristics of the clients receiving MMT

Characteristics	Total (*n* = 7181)
Gender, *n* (%)	
Males	5593 (77.9)
Age, *n* (%)	
15–24	175 (2.4)
25–44	1617 (57.2)
45–74	2900 (40.3)
Marital status, *n* (%)	
Never married/Single	4521 (63.0)
Married /Living together	2648 (36.9)
Others	12 (0.1)
Education, mean(SD)	
≤ middle school	4696 (65.4)
High school /Technical secondary school	2290 (31.9)
≥ college	195 (2.7)
Employment, n (%)	
Unemployed	5850 (81.5)

**Fig. 1:**
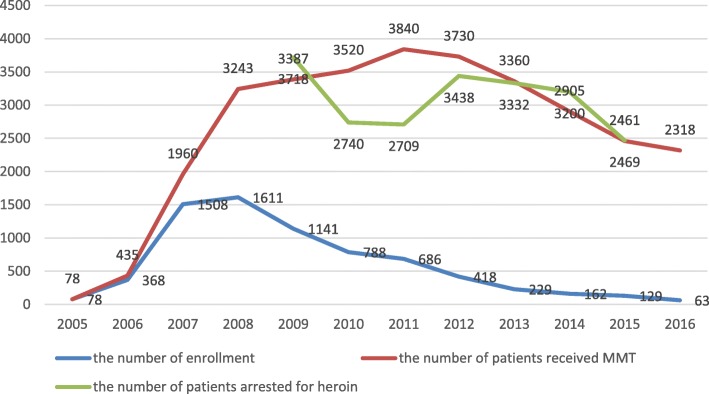
The number of patients received MMT, new enrollment, and arrested for heroin use

Fig. 1 presents the number of patients who received MMT each year and the number of new enrollment to MMT each year from 2005 to 2016. The number of patients receiving MMT per year increased sharply from 2006 to 2008, continued to increase gradually after that, and reached its peak with the number of 3840 in 2011. However, the number of patients receiving MMT per year has been declining gradually since 2012, and only 2318 patients received MMT in 2016. For the number of new enrollment each year, it showed an increasing trend from 2005 to 2008. However, the number of new enrolment has shown a steady decreasing trend from 2009 to the present. In addition, Fig. 1 also shows the number of people being arrested per year for heroin use from 2009 to 2015, which remained relatively stable over the years.

Based on the CDC data, we identified that the main reasons of dropout are the following: (1) discontinuing medication due to unknown reasons (25%), (2) criminal activities other than drug-related crimes (20%), (3) relapse to heroin use (16%), and (4) physical disease (10%). In addition, based on the CDC data from 2005–2016, the average daily dosage of methadone was 56 mg with the range 52–61 mg.

### Qualitative study

#### Demographics

In total, 9 service providers and 22 MMT patients participated in focus group discussions or in-depth interview. The service providers included 6 MMT staff, 2 social workers, and 1 administrator. The mean age of service providers was 47.4 years (standard deviation, SD, 13.3), and the average years of education were 15.6 (SD 2.9). Among MMT patients, 68.2% were male, and the mean age was 48.2 years (SD 9.4) with 10.2 years of education (SD 1.8). Five had terminated treatment from MMT clinics during the study period while the others were active patients in MMT clinics. The participants’ characteristics are shown in Table 2.

**Table 2:** Demographic characteristics and drug use history of the participants who attend qualitative study

Characteristics	Patients (*n* = 22)	Service providers(*n* = 9)
Gender, *n* (%)		
Males	15 (68.2)	4 (44.4)
Age, mean (SD)	48.2 (9.4)	47.4 (13.3)
Marital status, *n* (%)		
Never married/Single	13 (59.1)	------------
Married /Living together	9 (40.9)	------------
Years of education, mean(SD)	10.2 (1.8)	15.6 (2.9)
Duration of MMT (years), mean (SD)	5.5 (3.4)	8.5 (3.3)
Employment, *n* (%)		------------
Unemployed	13 (59.1)	------------
Time of drug use (months)	104 (43.7)	------------
Average age of initial drug use, mean(SD)	22.8 (10.4)	------------
Treatment times for withdrawal	7.7 (5.4)	------------
MKS	23.0 (3.2)	27.3 (4.4)

#### Attitudes towards and knowledge about MMT

Scores on the Methadone Knowledge Scale were low for both patients and service providers. The mean score for patients was 23 out of a possible 35, while the mean score for service providers was 27 out of 35.

Attitudes of patients towards MMT were both positive and negative. Some saw MMT as a positive replacement for opioids of abuse.

Using heroin made me bankrupt. Methadone is an appropriate treatment for me because of saving money, controlling cravings and helping me build self-confidence. Otherwise I might steal or rob others for money. (Participant 02, male, patient.)

I was afraid of being arrested because of heroin use before I attended the methadone program, but now I feel safe. (Participant 04, male, patient.)

Some patients interviewed expressed a negative opinion about MMT. “My friend told me that it is hard to stop using methadone once you become addicted, and persuaded me to use as little methadone as possible. My wife does not want me to come to the clinic. I asked staff about this, but they could not give me advice on the problem.” (Participant 07, male, patients; participant 09, male, patients)

MMT staff expressed their concern about their lack of knowledge about MMT.

In our hospital, all the doctors are required to rotate through MMT clinics in 2 year cycles, and there is no chance for them to attend an MMT training program. (Participant 25, female, MMT staff.)

Patients got more information about MMT. We know little about it. It is not enough to help them or persuade them stay in treatment. (Participant 30, female, social worker)

#### Factors affecting retention rate

Regarding the reasons for dropping out of MMT clinics, a patient cited the inconvenience of being in the clinic, poor services, and poor control of craving for opioids. In China, patients are required to take methadone on site. However, most clinics are only open during normal business hours when patients need to be at work. Some patients reported discontinuing methadone use due to fear of being terminated from their employment.

It is almost impossible for me to get one hour off to get methadone. Once my colleagues and my boss know that I am a drug user, I will lose my job. (Participant 13, female, patient)

Last year, I stayed in the hospital for two weeks because of leg surgery. My husband wanted to help me pick up methadone, but MMT staff said it is not allowed. I had to stop taking it for a week, then I went to the clinic every day by taxi from the hospital. It would have been easy to relapse at that time. (Participant 17, female, patient)

Both patients and MMT staff expressed that services offered by clinics could be expanded beyond just MMT to include health education, psychiatric care, or medical care. “Hard work, low salary, limited training, we could not provide more services to them.” (Participant 24, female, MMT staff)

It is difficult for me to communicate with drugs addicts since most of them have a co-morbid personality or mood disorder. (Participant 23, female, MMT staff)

#### Factors contributing to the decline in new enrollment in MMT in China

Both patients and staff agreed that the appearance of new synthetic drugs was the main reason for the decline of new enrollment in MMT. In addition, fear of being arrested and fear of developing a physical dependence to methadone were also cited as reasons for the decline in new enrollment in MMT.

Young people like to pursue excitement, so they would like to use methamphetamine or ketamine. We don’t like that feeling. Some heroin users’ change to using methamphetamine since this drug is easier to buy compared to heroin. Some heroin users do not want to be registered in the public security system, so if they want to use methadone, they prefer to buy it on the black market rather than going to a clinic. (Participant 31, male, MMT staff)

Stigma and concern about discrimination were noted by some patients. “My friend has used heroin for more than 10 years, but he refuses to attend the MMT program. He does not want his friends and relatives to know that he is a drug addict, and he does not want to be registered in the public security system.” (Participant 16, female, patient)

One of my friends was arrested when she left the clinic and was asked to take a urine test. It is not safe for drug addicts. (Participant 04, male, patient)

#### How to improve access to MMT

MMT staff suggested that it is important to increase awareness of the benefits of MMT among opioid users. They suggested that the MMT clinic serves as a comprehensive provider of other services, including education and medical care, and that this could attract more patients to MMT. In addition, they expressed that regular professional training regarding MMT and other topics related to substance use disorders could help them increase their self-confidence and allow them to help patients in the day-to-day work.

I have worked in MMT for almost 8 years, and I only got MMT training once at the beginning. When patients ask questions about their drug use problem, I still have no idea how to answer these questions. (Participant 24, female, MMT staff)

Provide more comprehensive service, including counseling, education and physical examination. As incentive, patients could take methadone out of the clinic based on the results of the urine test from the last week. The government should take measures to make drug addicts feel safe. (Participant 31, male, patient)

## Discussions

This was the first study to characterize the trends of patients receiving MMT from the beginning of its establishment to the present (2005–2016) in Shanghai, China. The number of MMT patients per year showed an increasing trend in the first 7 years but showed a gradual decrease after that. The new enrollment per year for MMT showed a similar pattern of change, which is consistent with the national trends as reported by the China CDC [11]. No previous studies have clarified the reasons for this change. We speculate that the sharp increase in the number of MMT patients in the earlier years might be due to the strong support from the government when the MMT just established in China. The decrease in the number of MMT patients might be due to a number of reasons. Both patients and service providers mentioned that the increase in ATS users may have driven the decline of MMT patients. However, the data from Shanghai Public Security Bureau indicated that the number of heroin user arrested per year remained stable over the years, suggesting that there might be other reasons other than the increased number of ATS users to account for the decreased number of MMT patients in recent years. Previous studies have identified several barriers to access the MMT, such as limited MMT knowledge, low methadone dosage, inconvenience of taking medicine, and financial burden [7, 12]. Consistent with the findings from the literature, our study has identified several major reasons, as discussed in detail below, to explain the decline in the number of MMT patients.

### Limited knowledge of MMT in both patients and service providers

Both patients and service providers had limited MMT knowledge in our qualitative study. Patients mentioned the fear of becoming methadone dependent and the preference of being on methadone “as little as possible.” A large body of research has demonstrated that a higher methadone dosage is more effective to promote treatment compliance and reduce heroin use, and under-dosing is one of the predictors for treatment dropout [7, 13, 14]. In the current study, the average methadone daily dosage was 56 mg, which is considered as a low dosage [13]. Moreover, the service providers also had limited knowledge about MMT and had similar misconceptions about the methadone dosage as in the patients.

### The staff’s perspective

In Shanghai, MMT clinics are affiliated to mental health institutions. Most psychiatrists are reluctant to work with drug users, who are considered by the general public as having moral defect. Staff members who are assigned to MMT clinics tend to be old and near the retirement age. Major complaints from the MMT staff include low salary, heavy workload, and limited MMT training. Many staff lack the motivation to provide high-quality service to MMT patients. This may also contribute to the decreased number of patients who remain in MMT.

### Complicated enrollment criteria

According to the Anti-Drug Law of the People’s Republic of China, drug users were defined as patients as well as offenders, so if a drug user is interested in MMT, he or she needs to register in the Ministry of Public Security system; the drug use history will become available to the general public. The patient may have difficulty to find a job and may lose access to various social benefits. Some drug users do not want to let the general public know their drug use history, and they prefer to have “cold turkey” withdrawal at home. Previous research has demonstrated that complicated enrollment criteria are an important factor that prevents drug users from participating in MMT [15, 16]. Policymakers should consider to simplify the enrollment criteria, and make the access to MMT programs easy in China.

As reported in other studies [17, 18], other barriers for patients to access MMT identified in our study include inflexible service hours, limited locations, and the restriction that patients only can take methadone within the clinic.

We acknowledge a number of limitations of this study. First, patients in our study were recruited from Shanghai MMT clinics, so the study sample may not be representative for patients in comparisons with those dropped out from treatment and other geographic areas. Secondly, there might be self-report bias in our study participants. Finally, there might be other barriers, which were not examined in our study, that prevent the implementation of MMT program in China.

## Conclusions

In summary, our study has identified various challenges and barriers to explain the decreased number of patients receiving MMT. Service providers, government agencies, patients and families, and other partners and stakeholders will need to work together to address these identified challenges and barriers, so more heroin users can benefit from MMT.

Future studies that include patients from other parts of China are needed to provide guidance on implementing MMT programs in China.

## Abbreviations

ATS: Amphetamine-type stimulants
CDC: Shanghai Centers for Disease Control
HIV: Human immunodeficiency virus
MKS: Methadone Knowledge Scale
MMT: Methadone Maintenance Treatment
SD: Standard deviation
SPSS: Statistical Package for the Social Science
